# Lessons on Mass Gatherings Learned From the 2019 Union Cycliste Internationale Mountain Bike World Cup

**DOI:** 10.7759/cureus.14275

**Published:** 2021-04-03

**Authors:** Paul Craven, Joseph Hansroth, Kimberly D Quedado, Christopher S Goode, Shane Dragan, Aaron Monseau, Brenden Balcik, Nicholas Chill, Scott W Findley

**Affiliations:** 1 Emergency Medicine, West Virginia University School of Medicine, Morgantown, USA

**Keywords:** mountain biking, event medicine, emergency medicine, wilderness medicine, international sporting events

## Abstract

The Union Cycliste Internationale (UCI) Mountain Bike World Cup in 2019 provided unique challenges for effective prehospital care. While on-site medical care has demonstrated improved outcomes along with reduced emergency department and emergency medical services (EMS) utilization, this aspect has not been well documented in the literature with respect to rural mass gathering events (MGEs). Conducted at a large mass gathering event in a geographically isolated area, this study aimed to assess the medical needs at this specific event and will hopefully assist in future coordination of similar events.

All patients who were treated at the event clinic were included in the analysis. Primary investigators collected and recorded data while providing care. We believe the on-site clinic was successful in reducing barriers to healthcare by improving access, streamlining the treatment process, and optimizing resource utilization. This benefit extended to race participants, support staff, spectators, and the local EMS system.

## Introduction

Why this is unique

There has been an increase in mass gathering events (MGEs) in remote areas secondary to an increase in participation in adventure sports [1]. Although these elite athletes are typically young and healthy, there is an increased risk of injury among them due to the nature of the events [2,3]. On-site medical care serves a critical role in achieving improved outcomes along with reduced emergency department and emergency medical services (EMS) utilization [4]. However, the provision of high-quality medical care in an austere environment often presents its own unique challenges and demands. While mountain bike injuries and large MGEs-related care have been documented, there is scarce data available in the literature related to the combination of rural MGE and mountain bike medical care. The goal of this study was to document the care deployed at a mass gathering at a mountain biking event held in a remote area of West Virginia (WV).

Location

The Union Cycliste Internationale (UCI) Mountain Bike World Cup 2019 was held in Snowshoe, WV in September 2019. Snowshoe is located in the eastern mountains of West Virginia within the Green Bank National Radio Quiet Zone (NRQZ,) which is home to National Radio Astronomy Observatory and requires a restriction on all radio waves within a 13,000 mi^2^ area [5]. Communications, including cellular services, is limited in this geographical region. The area is home to a single critical access hospital (CAH) with a seven-bed emergency department, which is located approximately 45 minutes away.

Event scope

The UCI event drew 374 participants with an estimated 10,000 support staff and spectators to the area [6]. It consisted of four days of practice runs, qualifying trials, and active racing. Both males and female athletes competed in the event and were further subdivided into adult- and junior-age groups. The event included a downhill competition and cross-country competitions comprising long- and short-track events. The downhill course was 2 km long with steep descents, multiple terrain features, and large obstacles. The majority of the course was difficult to access for spectators and support staff, often requiring the use of support railings and ropes to navigate. The cross-country course was 34 km long, while the short-track course was 9 km in length. The average downhill speed for the male elite riders was 40 km/hour with a top speed of 57 km/hour.

Clinical support

A volunteer medical team from the academic emergency department at West Virginia University (WVU) provided medical services at the event. This team of providers established an on-site medical clinic immediately adjacent to the racecourses. Providers were scheduled to work in shifts pairing attending physicians with a sports medicine fellow, emergency medicine resident, or an emergency medicine advanced practice provider (APP) to provide care either in the clinic or in the mountains in coordination with the course bike patrol. The overall volunteer crew consisted of six attending physicians, two sports medicine fellows, three emergency medicine residents, and two emergency medicine APPs. When they were not staffing the tent, the providers served as monitors along the course. The medical tent was stocked with a variety of medical equipment and safety tools. This included orthopedic equipment such as Ortho-Glass®, splints and slings, suture kits, resuscitative medications, and extrication equipment.

In preparation for this MGE, the volunteer team had focused on fulfilling the strategic care needs including placement of providers on the scene, point of care ultrasound (POCUS), and coordination with the event bike patrol who operate the clinic year-round, local EMS, and aeromedical agencies [7]. The optimal model of medical support at MGEs relating to rural adventure sports is still evolving, and this study sought to assess the general staffing and resource needs at the UCI 2019 event to gain an understanding of the planning and preparedness required at similar MGEs in the future conducted in austere geographical settings [1].

## Materials and methods

Study design and participants

This observational study was reviewed by the WVU Institutional Review Board (IRB) and was approved under a FLEX designation protocol (non-federally funded, minimal-risk IRB submissions). With the exception of any protected health information, every patient encounter at the centralized event clinic was included in the analysis and documented on-site. All data was immediately entered into a Microsoft Excel (Mac 2016 version 16.42) and charts were completed at the time of patient encounter. Specific metrics collected consisted of basic demographic information including age, sex, participant type, country of origin, nature of the injury, treatment modalities used, disposition with recommended follow-up, mechanism of transport, and instances of refusal of treatment. Injuries were described based on presenting mechanism in trauma patients, stability of the patient, medical condition, or if the visit was related to the follow-up of a prior injury or illness. Treatments were categorized based on the need for reduction and splinting, regional anesthesia, intravenous (IV) fluids, medication, and wound care. The incidence of POCUS utilization was recorded and subcategorized as diagnostic- or treatment-related. Descriptive statistics were calculated in Excel based on the collected data.

## Results

A total of 42 patients were treated at the clinic over the four-day event (Table 1). Their ages ranged from 10 to 66 years, with 21 (50%) patients being from the United States (US) and the remaining 21 patients representing other countries. Of the 347 race participants, 25 (7.2%) participants presented to the clinical tent. The non-competitor presentation included five support staffers and 12 spectators from the estimated 10,000 non-competitor attendance at the event. Four of the 42 (10%) patients presented for follow-up care for injuries sustained at prior events.

**Table 1:** Patient demographics

Variables	Patient type			
	Injured race participants, N=25	Injured support staff, N=5	Injured spectators, N=12	Total injured patients, N=42
Sex				
Female, n (%)	7 (28%)	1 (20%)	3 (25%)	11 (64%)
Male, n (%)	18 (72%)	4 (80%)	9 (75%)	23 (32%)
Age in years				
Average	23	34	29	29
Range	16-36	19-47	10-66	10-66
Standard deviation	5.2	10	17.6	12.9
US citizens, n (%)	5 (20%)	5 (100%)	11 (92%)	21 (50%)

The majority of injuries were musculoskeletal in origin, representing 32 (76%) of 42 patients who presented for care (Table 2). Thirteen of the 42 (31%) patients presented with injuries concerning for fracture and/or dislocation. Final diagnosis beyond our on-site clinical impression was not available for all patients.

POCUS was the primary imaging modality as no other imaging modality was available on-site. In total, POCUS was used to evaluate 17 of 42 (40%) patients in some manner as either a screening, diagnostic, or procedural tool. This included two guided regional nerve blocks.

**Table 2:** Evaluation and treatments

	Total patients (N=42)	Percentage of total injured patients
Interventions		
Evaluation only	12	29%
Evaluation and ultrasound	17	41%
Evaluation and treatment	30	71%
Intravenous fluids	1	2%
Wound care	18	43%
Immobilization/splint	8	19%
Sling	8	19%
Regional anesthesia	4	10%
Ultrasound use		
Focused assessment with sonography in trauma	4	10%
Anesthetics	2	5%
Fractures	11	26%
Joint/soft tissue	4	10%
Testicular	1	2%
Clinical impressions		
Suspected fracture	12	29%
Dislocation	2	5%
Testicular torsion	1	2%
Abrasions	7	17%
Laceration	4	10%
Concussion	1	2%
Concussion reevaluation	2	5%
Suture removal	1	2%
Wound closure	1	2%
Pilonidal cyst packing	1	2%
Migraine	1	2%
Blister	1	2%
Burn	1	2%
Anaphylaxis	1	2%
Intracranial hemorrhage	1	2%
Dehydration	1	2%
Rib pain	2	5%
Sprain/joint pain	6	14%

All patients, with the exception of a spectator who was evacuated directly from the scene, were seen in the clinic and dispositioned to home or hospital via private vehicle (Table 3). Of the 42 patients seen, 33 (79%) patients were discharged home with outpatient follow-up. Nine (21%) patients required transfer to a higher level of care. Four (44%) of the nine transferred patients were referred to the local CAH, while five (56%) were determined to require evaluation at larger medical institutions. Seven (16%) refused some level of care with two (<1%) refusing recommended interventions citing that it would impair their ability to compete. Of the seven who refused care, six (86%) were non-US citizens and preferred to follow up in their home country.

**Table 3:** Disposition of patients

Disposition	Total patients (N=42)	Percentage of total injured patients
Home/self-care	33	78
Critical access hospital	4	10
Regional hospital	4	10
Regional level 1 trauma center	1	2

## Discussion

Several studies have been conducted to assess mountain biking injuries and pathology, ranging from assessment of overall trauma to increased risk of testicular pathologies [2,8-12]. Prior studies have also assessed the potential benefits of having physicians and APPs on the scene for large events to help improve the quality of care [7,13,14]. Our study was built off this prior framework, highlighting the unique challenges in supporting an MGE in a remote wilderness location.

As anticipated, the majority of injuries were traumatic, musculoskeletal in nature, and race participants accounted for the highest rate of injured patients. Presentation for medical care was split, with 60% of the presenting patients being racers and 40% non-competitors. Presentation, management, and treatment of predicted injuries were discussed in depth by the medical team prior to the first day. Supplies to address these injuries were obtained prior to the departure of the crew or confirmed to be available at the mountain clinic. During the event, a number of non-traumatic needs became evident with patients seeking care for medical conditions including complex migraine, pilonidal cyst, anaphylaxis, and viral upper respiratory infections (URIs). Visits for follow-up care represented 10% of patient encounters, which was unanticipated, including individuals presenting for follow-up for concussions and lacerations suffered at prior races, requiring reassessment and suture removal. Based on the higher-than-anticipated volumes of patients presenting for follow-up care from prior events, the authors would recommend stocking additional medical supplies such as suture removal kits and over-the-counter medications for similar future events.

The mountain clinic was stocked with medications and materials to treat traumatic injuries; however, it did not have on-site diagnostic imaging capability. The medical team anticipated the need for imaging and obtained a portable ultrasound machine prior to the event. Throughout the event, POCUS provided advanced diagnostics, and it was used to perform a focused assessment with sonography in trauma (FAST) exam in four cases to assess for obvious hemorrhage. POCUS was also utilized for orthopedic evaluations of fractures and dislocations in 13 cases, which involved instances such as a clavicle fracture confirming the diagnosis. Furthermore, treatment of these injuries was facilitated using POCUS to provide effective regional anesthesia such as intra-articular injection to facilitate closed shoulder reduction and hematoma block for distal radius fracture. In the absence of on-site diagnostic imaging in such austere environments, healthcare providers who are well-versed in POCUS are able to provide a higher level of care through early diagnosis of acute pathologies, as shown by the injuries identified during this event. In one instance, an ultrasound fellowship-trained physician's clinical impression led to a confirmed diagnosis of testicular torsion using POCUS. This resulted in a transfer for definitive surgical care.

In the absence of this provider clinic, patients would have been advised to be transported by EMS or referred to seek care at the closest hospital by private transport. Based on patient presentations, we believe numerous patients would have required transport by EMS, which would have put a strain on the local EMS system given the increased volume of transports and remote location during the four-day event. Thus, the establishment of the on-site medical clinic streamlined care by triaging patients to those who could be treated on-site versus those who required stabilization and transport to a higher level of care. This paper would propose that coordination with local EMS prior to the event can optimize staffing and apparatus needs for local prehospital care personnel.

There were several unanticipated barriers to providing healthcare during the event. This particular weekend witnessed the last race of the season and it was the finals for the season-long quest for the UCI World Cup. Some racers refused interventions on the basis that it would impair their ability to compete, and upper extremity splinting was declined by two racers as a result. Several competitors, especially international competitors, were hesitant to seek care due to concerns of cost and a desire to follow up in their home countries. While not quantified, some competitors changed their willingness to present to the clinic when they learned the services provided by volunteer staff were free of cost.

Limitations of the study

There were some limitations due to the novelty of the event. No data were obtained for anyone who may have bypassed the event clinic and presented directly to a hospital. Therefore, it is unknown as to how many participants presented directly to outside care facilities to receive evaluation and treatment. There are also concerns that competitors may not have presented to the clinic out of fear of being told they could not compete anymore. The study was not able to gauge the competitors' level of access to healthcare outside of their assessment at this event. Many patients were referred to care facilities in the vicinity or recommended to follow up with their primary sports medicine provider for additional care, making it difficult for the authors to follow up on final diagnoses and subsequent medical needs (i.e., surgical interventions). Additionally, as many of the participants were professional athletes with some being international, it is unknown as to how many eventually followed up on the recommendations of the on-site clinic providers.

## Conclusions

The presence of physicians and APPs on the scene for major events can provide specialized care in terms of diagnosis and treatment. At the event that we discussed in this study, practitioners on-site provided fracture and dislocation reductions, anaphylaxis management, and regional anesthesia, which would not have been otherwise available. The use of POCUS allowed for rapid trauma assessments through FAST exams, confirmation of fractures, and evaluation of medical conditions such as testicular torsion. While many of these patients required follow-up, the level of urgency and resource requirement, including the burden on the local EMS system, was greatly reduced. We believe the on-site clinic was successful in reducing barriers to healthcare in such an austere environment by improving access, streamlining the evaluation and treatment process, and optimizing resource utilization. This benefit extended to race participants, support staff, spectators, the local EMS system, and the regional CAH because of our initial triage. Future studies would benefit from assessing patient follow-ups from on-site care provided at MGEs, health insurance status, and other barriers to healthcare unique to a remote international event.

## References

[1] Laskowski-Jones L, Caudell MJ, Hawkins SC (2017). Extreme event medicine: considerations for the organisation of out-of-hospital care during obstacle, adventure and endurance competitions. Emerg Med J.

[2] Becker J, Runer A, Neunhäuserer D, Frick N, Resch H, Moroder P (2013). A prospective study of downhill mountain biking injuries. Br J Sports Med.

[3] Ansari M, Nourian R, Khodaee M (2017). Mountain biking injuries. Curr Sports Med Rep.

[4] Goldberg SA, Maggin J, Molloy MS (2018). The Gillette Stadium experience: a retrospective review of mass gathering events from 2010 to 2015. Disaster Med Public Health Prep.

[5] Green Bank Observatory (2021). Green Bank Observatory: National Radio Quiet Zone. https://greenbankobservatory.org/about/national-radio-quiet-zone/.

[6] Mercedes-Benz UCI Mountain Bike World Cup (2019). Mercedes-Benz UCI Mountain Bike World Cup. http://www.uci.org/mountain-bike/events/mercedes-benz-uci-mountain-bike-world-cup.

[7] Grange JT, Baumann GW, Vaezazizi R (2003). On-site physicians reduce ambulance transports at mass gatherings. Prehosp Emerg Care.

[8] Mitterberger M, Pinggera GM, Neuwirt H (2008). Do mountain bikers have a higher risk of scrotal disorders than on-road cyclists?. Clin J Sport Med.

[9] Bush K, Meredith S, Demsey D (2013). Acute hand and wrist injuries sustained during recreational mountain biking: a prospective study. Hand (N Y).

[10] Chow TK, Kronisch RL (2002). Mechanisms of injury in competitive off-road bicycling. Wilderness Environ Med.

[11] Hurst HT, Atkins S, Dickinson BD (2018). The magnitude of translational and rotational head accelerations experienced by riders during downhill mountain biking. J Sci Med Sport.

[12] Stoop R, Hohenauer E, Vetsch T, Deliens T, Clijsen R (2019). Acute injuries in male elite and amateur mountain bikers: results of a survey. J Sports Sci Med.

[13] Moreno Millán E, Bonilla F, Alonso JM, Casado F (2004). Medical care at the VIIth International Amateur Athletics Federation World Championships in Athletics 'Sevilla '99'. Eur J Emerg Med.

[14] Janicke DM, Jacob DJ, LaFountain RB, Pundt MR, Young GE (1995). Emergency medical care in the athletes' village: World University Games 1993. Prehosp Disaster Med.

